# Focus on the Visible Regions: Semantic-Guided Alignment Model for Occluded Person Re-Identification

**DOI:** 10.3390/s20164431

**Published:** 2020-08-08

**Authors:** Qin Yang, Peizhi Wang, Zihan Fang, Qiyong Lu

**Affiliations:** Key Laboratory for Information Science of Electromagnetic Waves, Research Center of Smart Networks and Systems, School of Information Science and Technology, Fudan University, Shanghai 200433, China; 18210720046@fudan.edu.cn (Q.Y.); 17210720042@fudan.edu.cn (P.W.); 19210720032@fudan.edu.cn (Z.F.)

**Keywords:** deep learning, person re-identification, occlusion, semantic segmentation, feature fusion

## Abstract

The occlusion problem is very common in pedestrian retrieval scenarios. When persons are occluded by various obstacles, the noise caused by the occluded area greatly affects the retrieval results. However, many previous pedestrian re-identification (Re-ID) methods ignore this problem. To solve it, we propose a semantic-guided alignment model that uses image semantic information to separate useful information from occlusion noise. In the image preprocessing phase, we use a human semantic parsing network to generate probability maps. These maps show which regions of images are occluded, and the model automatically crops images to preserve the visible parts. In the construction phase, we fuse the probability maps with the global features of the image, and semantic information guides the model to focus on visible human regions and extract local features. During the matching process, we propose a measurement strategy that only calculates the distance of public areas (visible human areas on both images) between images, thereby suppressing the spatial misalignment caused by non-public areas. Experimental results on a series of public datasets confirm that our method outperforms previous occluded Re-ID methods, and it achieves top performance in the holistic Re-ID problem.

## 1. Introduction

Person re-identification (Re-ID) is a task that aims to retrieve a given pedestrian in a cross-camera system; it can be combined with pedestrian detection and pedestrian tracking technology and applied in video surveillance, intelligent security and other fields. In recent years, deep learning technology has been widely used in person Re-ID tasks and achieved great progress [1,2,3,4,5,6,7].

However, in realistic situations, pedestrians are often occluded by cars, buildings or trees. This occlusion problem greatly affects the retrieval performance of the pedestrian re-identification task. However, most previous methods [1,2,3,4,5] do not consider this problem. Compared with the holistic person Re-ID, the occluded person Re-ID presents two unique challenges. 

**Figure 1:**
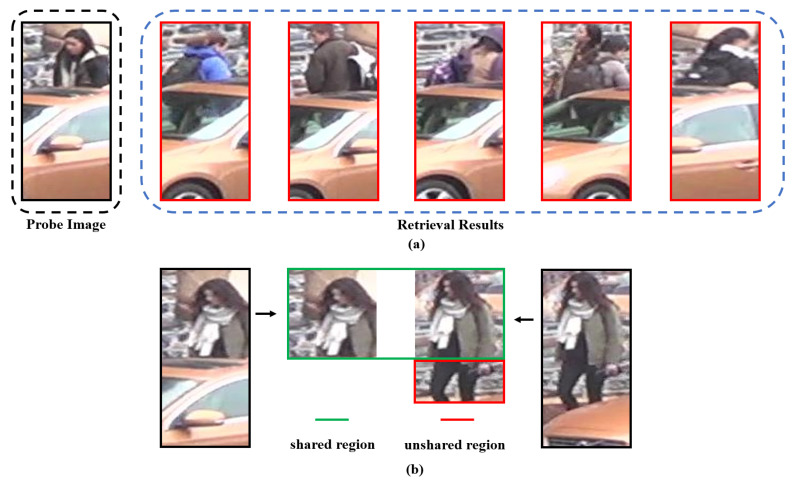
Two challenges of the occluded person re-identification problem. In (**a**), owing to the influence of obstacles, previous Re-ID methods may incorrectly retrieve the image of a similar car; in (**b**), the unshared regions (the red region on the right image) become distracting noises that interfere with retrieval.

First, owing to the occluded part of the image, the extracted features are disturbed by obstacles, which seriously affect the retrieval results. As shown in Figure 1a, a pedestrian is occluded by a car, and previous methods mixed the information of the car with the features of the person and erroneously retrieve images of a similar car.

Second, even if we only focus on the visible parts of persons as a result of different occlusion conditions, the unshared body regions of two images become distracting noises instead of discriminative clues: as shown in Figure 1b, the right person’s legs have become distracting noises during the retrieval process. 

Some recent works [8,9,10,11,12] have tried to solve the occlusion problem and call it the partial Re-ID problem. In this problem, all gallery images are holistic images, and all probe images are manually cropped to preserve the visible parts. There are two problems found in the partial Re-ID problem: (1) it supposes that in the gallery images, no pedestrians are occluded by obstacles, but this is not always consistent with the real world; (2) they manually crop all occluded images, and when there are many occluded images in a retrieval task, this work is inefficient and unrealistic.

**Figure 2:**
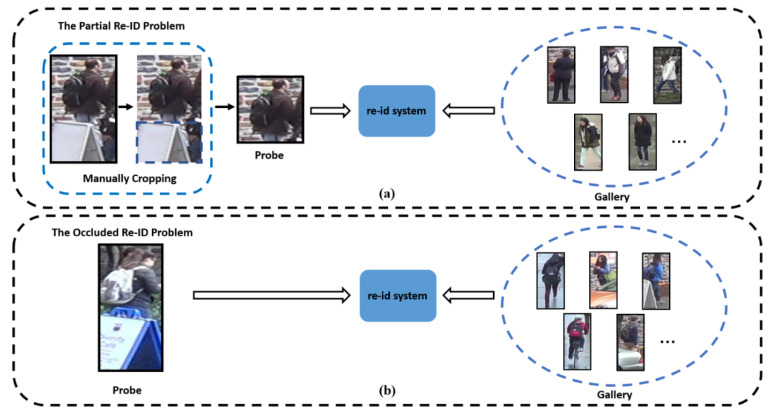
Comparison of the partial re-identification problem and the occluded re-identification problem. In (**a**), all probe images are manually cropped, leaving only the visible regions of pedestrians, and all gallery images are holistic. In (**b**), all probe images contain obstacles, and the gallery contains both occluded and holistic images.

In view of the limitations of the partial Re-ID problem, the authors of [12] proposed the occluded Re-ID problem. As shown in Figure 2b, in this problem, apart from the probe set, there also exist occluded images in the gallery set, and all occluded images retain their occluded regions. Therefore, the occluded Re-ID problem is closer to the real-world situation. In this article, we study the occluded Re-ID problem.

To deal with this more difficult occluded Re-ID problem, we propose a semantic-guided alignment model (SGAM), which uses human semantic information to guide the network to focus on the visible area of images and learn region-level features. To be more specific, we first train a human semantic parsing network: the input is an occluded image, and the network outputs semantic probability maps of different human regions. These probability maps perceive which regions are visible and instruct the model to automatically crop occluded areas of images. In the feature construction stage, SGAM fuses the probability maps and global features to extract local features of visible areas. In the retrieval phase, SGAM focuses on the public visible areas between images and calculates the distance of corresponding features, thereby determining the similarity of the images.

SGAM provides a 3-fold benefit in improving occluded Re-ID’s performance: (1) in the preprocessing stage, in a completely automatic step, SGAM separates the occluded area from images and only retains the visible area, thus avoiding noises in the occluded area; (2) in the feature construction stage, SGAM extracts local features of visible areas through semantic information: it benefits from fine-grained information, and features have higher discrimination capabilities; (3) in the matching stage, SGAM only calculates the shared visible region’s local distances between images, thus suppressing the distracting noises caused by unshared regions and spatial misalignment.

The main contributions of this paper are summarized as follows:

- We propose an automatic cropping method for the occluded Re-ID problem. It can automatically crop the occlusion regions of pedestrian images and retain the visible regions, avoiding the inefficiency and human bias of manual cropping. This method can be embedded in any occluded Re-ID model;
- We propose a semantic-guided alignment model (SGAM), which can use semantic information to guide the model to extract local features in pedestrians’ visible regions and to only focus on the public visible areas between images during the matching stage, thus significantly suppressing interference noises caused by spatial misalignment and unshared regions;
- We conducted several experiments on a series of public Re-ID datasets [9,12,13,14,15,16] to verify the effectiveness of SGAM. Experimental results demonstrate that our model outperforms previous occluded Re-ID methods [8,9,10,11,12]. In the holistic Re-ID problem, our method still achieves competitive performance. Sufficient ablation experiment results confirm that SGAM has outstanding matching capability, and the proposed strategies can be easily embedded into other pedestrian re-identification methods.

The rest of this paper is organized as follows. Related work is reviewed in Section 2. We introduce the proposed SGAM method in detail in Section 3. In Section 4, we present extensive experimental results, and we conclude this paper in Section 5.

## 2. Related Work

### 2.1. Deep Person Re-Identification

In recent years, pedestrian re-identification methods based on deep learning have significantly improved retrieval accuracy. Recent works [2,17,18] show that combining local features of body parts can construct a more efficient representation. For example, Sun et al. [2] split the feature map uniformly and used multiple classifiers to learn part-level features. Wei et al. [17] employed pose landmarks to extract local features of pedestrians and to connect these features for retrieval. Wang et al. [18] partitioned the images into several stripes, and the model learned fine-grained features and combined global features to form multi-scale descriptors.

However, previous works have had difficulties when dealing with the occluded Re-ID problem. As shown in Figure 1, when images are occluded, previous methods mix the information of occluded regions and visible regions in the final feature representation, thus greatly reducing the retrieval accuracy.

### 2.2. Partial Person Re-Identification

Recently, some works have been dedicated to solving the partial Re-ID problem [8,9,10,11,12]. He et al. [8] proposed a matching model based on sparse reconstruction learning and called it deep spatial feature reconstruction (DSR). DSR can automatically match images of different sizes, thus avoiding the time-consuming spatial alignment step. He et al. [10] further proposed a spatial feature reconstruction model, which generates multi-scale features by a fully convolutional network to deal with the scale change of feature maps. Zheng et al. [9] proposed a local matching strategy based on dictionary learning and called it ambiguity-sensitive matching classifier (AMC), and they introduced a sliding window matching (SWM) model to solve global-part-based matching problems. Sun et al. [11] proposed a visibility-aware part model (VPM), which perceives visible areas by self-supervised learning to avoid the noise effect of the occluded regions. Miao et al. [12] proposed a pose-guided occluded Re-ID method, which exploits pose landmarks to measure the visibility of regions and extracts local features of the visible region for retrieval.

However, in [8,9,10,11], only the probe set contains occluded images, and all occluded images are manually cropped to preserve the visible parts. This is different from real-world scenarios. Therefore, we consider a more general situation: some gallery images are occluded, and all occluded images retain their occluded regions: this is the occluded Re-ID problem mentioned above.

### 2.3. Semantic-Guided Person Re-Identification

Recently, some works [19,20,21] have employed semantic information in person Re-ID models. Cheng et al. [20] used a graphical structure to parse pedestrians into semantic information and extract local features, and Kalayeh et al. [19] employed a semantic segmentation network to extract multiple regional features of pedestrians. Zhang et al. [21] proposed a densely semantically aligned model that uses fine-grained semantic information to deal with the misalignment problems of images. 

However, as in the approaches in Section 2.1, these methods focus on the holistic Re-ID problem, and our work uses semantic information to deal with the occluded Re-ID problem. Through semantically guided attention mechanisms, our model focuses on the visible regions of images and extracts region-level features, thereby suppressing the noise impact of the occluded regions.

## 3. Materials and Methods

This section illustrates our proposed semantic-guided alignment model (SGAM), which consists of an automatic cropping strategy, semantic-guided feature extraction network and public distance measurement strategy.

### 3.1. Automatic Cropping Strategy

**Figure 3:**
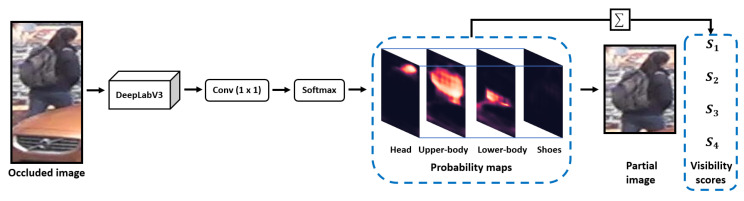
The structure of the automatic cropping strategy, whose main body is a human semantic parsing network, and the output probability maps guide the network to automatically crop the image.

The architecture of the automatic cropping strategy is shown in Figure 3. It is a human semantic parsing network, whose main body is the DeepLabV3 network [22]. It takes occluded images as the input and outputs probability maps associated to four different body regions, namely head, upper-body, lower-body and shoes, and the network calculates visibility scores corresponding to each probability map to determine which areas are visible.

The human semantic parsing network classifies each pixel in the image to generate four semantic probability maps, and these maps reveal the degree of occlusion of each area. The classification function used by the network is as follows:

(1) $$P\left(R_{i}|g\right)=softmax\left(W^{G}g\right)=\frac{expW_{i}^{G}g}{\sum_{j=1}^{p}W_{j}^{G}g},$$

where ***G*** represents the image tensor that is input into the human semantic parsing network, $R_{i}$ represents the i-th region, $P\left(R_{i}|g\right)$ is the probability that the pixel g belongs to $R_{i}$, W is the weight matrix learned by the 1 × 1 convolutional layer, and p is the number of human body regions (the default is 4).

It can be observed in Figure 3 that when the image has occlusion, the $P\left(R_{i}|g\right)$ of pixels in the corresponding area becomes low. By calculating the probability visibility score S, we can determine whether there is occlusion in the corresponding area, which is formulated by

(2) $$S_{i}=\underset{g\in G}{\sum }P\left(R_{i}|g\right),$$

Naturally, if the $S_{i}$ of the corresponding area is large, it reveals that the i-th area of the image is visible. In contrast, if the corresponding human foreground is occluded in the area, $S_{i}$ will be very small, and the network will crop the area. Through this strategy, SGAM can automatically crop the occluded regions in images and retain the pedestrian’s visible area.

### 3.2. Semantic-Guided Feature Extraction Network

Although the occluded regions are removed from the cropped images, the spatial misalignment problem illustrated in Figure 1 remains. To solve it, we propose a semantic-guided feature extraction network.

**Figure 4:**
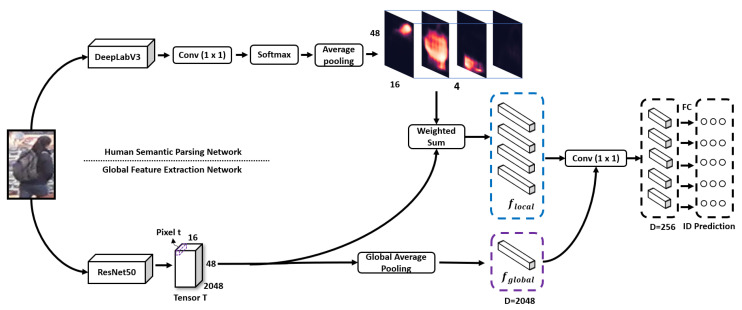
Semantically guided feature extraction network, which is composed of two branch networks: they are the human semantic parsing network and global feature extraction network. A cropped image is input, and the human semantic parsing network outputs four semantic probability maps, while another branch outputs the global feature of the image. The global feature and probability maps are merged by the dot product to extract four local features (head, upper-body, lower-body and shoes). Five features (four local features and one global feature) are trained by cross-entropy loss.

The architecture of the semantic-guided feature extraction network is shown in Figure 4. It is a two-branch architecture: one is a global feature extraction network, and the other one is the human semantic parsing network proposed in Section 4.1.

The global feature extraction network uses ResNet50 [23] as the backbone architecture and removes the average pooling layer and the fully connected layer. At the same time, we modified the stride of conv4_1 to 1 to get a larger feature map. A larger feature map can preserve more spatial information, which is beneficial for subsequent local feature extraction.

After the modifications above, the network outputs a feature map that is 4 times larger than that of the original ResNet50 [23]. We denote it as $T\in R^{h\times w\times c}$, where *h*, *w* and *c* denote the height, width and channel number of feature maps. 

To extract local features, we fuse the global feature map T with probability maps to extract four local features. More precisely, we first average pool the probability maps to match the size of the tensor T. Then, we apply weighted pooling to the global feature map with four resized probability maps, one for each global feature, and guide the network to focus on local visual cues, which is formulated by

(3) $$f_{i}=\frac{\sum_{t\epsilon T}AP\left(P\left(R_{i}|g\right)\right)t}{S_{i}},\forall i\epsilon 1,2,\ldots ,p,$$

where $AP\left(P\left(R_{i}|g\right)\right)$ represents the average pooling operation of $P\left(R_{i}|g\right)$, and t is the 2048-dim vector on the tensor T. Through weighted pooling, the network outputs four regional-level features, each of which represents one specific human body region. Under the guidance of semantic probability maps, each region-level feature retains the information of visible human body regions while ignoring the information of the background and other body regions, thus suppressing the influence of spatial misalignment on local feature extraction.

According to [18], combining multi-granularity information can obtain more discriminative features, so the model uses five features for training, including four local features and one global feature. Five feature vectors are fed into a 1 × 1 convolutional layer to reduce the dimension of vectors from 2048 to 256. Finally, the network inputs each feature vector to a softmax layer and uses cross-entropy loss for training. We use a special training strategy to train the network, which is illustrated in Section 3.4.

### 3.3. Public Distance Measurement Strategy

After solving the problem of the occlusion area and local feature extraction, the model faces the challenge of non-public area noise described in Figure 1. In order to make the SGAM concentrate on the public visible areas of images during retrieval, we propose a public distance measurement strategy.

For any input image, the SGAM always outputs four local features. Due to the presence of occlusion areas, some local areas are not visible. The common distance measurement strategy uses the visibility score $S_{i}$ proposed in Section 3.1 to weigh the contribution of each feature to the overall distance. More precisely, given two images $I_{m}$ and $I_{n}$ to be compared, the SGAM first calculates their region-to-region Euclidean distances $D_{i}^{mn}=∥f_{i}^{m}-f_{i}^{n}∥$, and then the SGAM uses $S_{i}$ to calculate the weight of the local feature distance $D_{i}^{mn}$ in the overall distance. The formula is as follows:

(4) $$D^{mn}=\frac{\sum_{i=1}^{p}S_{i}^{m}S_{i}^{n}D_{i}^{mn}+D_{global}}{\sum_{i=1}^{p}S_{i}^{m}S_{i}^{n}+1}$$

where $D_{global}=∥f_{global}^{m}-f_{global}^{n}∥$. In Equation (4), the visibility score $S_{i}$ controls the contribution of the local distance $D_{i}^{\mathrm{mn}}$ to the overall distance, so the local distance corresponding to the public visible area contributes more to the overall distance. In contrast, when a certain human body region is invisible in both images, because of the constraint of visibility scores, their local distance has little effect on $D^{mn}$. Through this strategy, the SGAM greatly reduces the impact of noise in non-public areas.

### 3.4. Training SGAM

**Figure 5:**
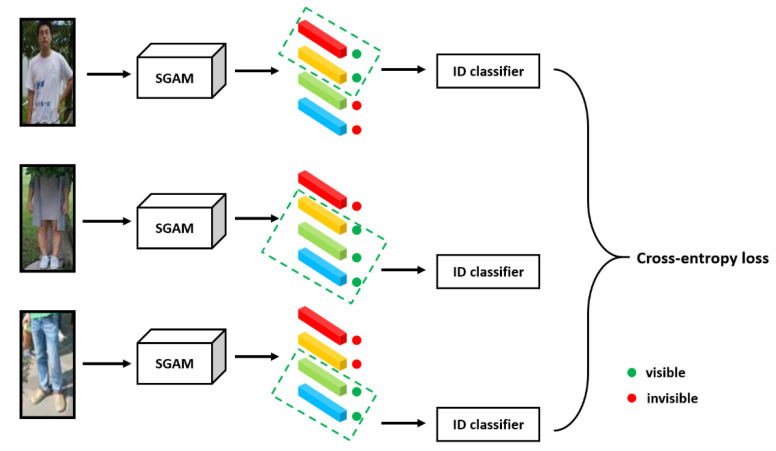
Semantic-guided alignment model (SGAM) training process. The model judges the visibility of each area through the visibility score, and only the image visible regions can cause loss.

Since the input images of the SGAM are cropped images, some human parts may be missed. As shown in Figure 5, the three input images lack the upper body, head and lower body regions, respectively. We want the SGAM to focus on the visible regions of input images during training. Following [11], we set some special training strategies for the SGAM.

As shown in Figure 4, for any input images, the SGAM always outputs four local features. However, during training, many human parts of the input image are missing, so we should only allow features in the visible area to cause training loss. Through the visibility score calculated in Equation (2), we can evaluate the visibility of each local area and dynamically select the visible area for feature learning.

Specifically, as shown in Figure 5, for each region, the SGAM sets a visible threshold. When the visibility score of the corresponding region is lower than the threshold, the SGAM regards the region as not visible in the image, and the cross-entropy loss caused by these local features is not counted in the total loss. The loss function of local features is as follows:

(5) $$L_{part}=-\sum_{i=1}^{p}max\left(S_{i}-t_{i},0\right)log\left(softmax(IP_{i}\left(f_{i}\right))\right)$$

where $t_{i}$ is the visible threshold corresponding to the region feature $f_{i}$. $IP_{i}\left(f_{i}\right)$ represents a feature classifier, which uses feature $f_{i}$ to predict the identity of the training images. Through Equation (5), the model focuses on the visible regions and learns the region features.

Similarly, the loss function for the global feature is as follows:

(6) $$L_{global}=-log\left(softmax(IP\left(f_{global}\right))\right)$$

Therefore, the final loss function is

(7) $$L=\lambda L_{global}+\left(1-\lambda \right)L_{part}$$

Among these variables, λ is used to weigh the contribution of $L_{global}$ and $L_{part}$ in the final loss. In Section 4.3, we discuss the impact of λ on model performance.

## 4. Results

### 4.1. Datasets and Evaluation Measures

To verify the performance of the SGAM, we evaluated it on several public datasets, including Occluded-DukeMTMC, Partial-REID, Partial-iLIDS, Market1501 and DukeMTMC-reID. 

Occluded-DukeMTMC [12] is re-segmented from the original DukeMTMC-reID dataset. The training set contains 15,618 images, and the gallery set and query set contain 17,661 and 2210 images, respectively, in which all query images and some gallery images are occluded images, and these occluded images retain their occluded regions without being manually cropped. Occluded-DukeMTMC was used to verify the efficacy of the SGAM in the occluded Re-ID situation.

The Partial-REID [9] dataset contains 600 images of 60 people, and each person has 10 images, including 5 occluded images and 5 holistic images. In contrast to the Occluded-DukeMTMC dataset, these occluded images are pre-cropped and only retain the visible parts.

Partial-iLIDS [15] is a partial Re-ID dataset based on the iLIDS dataset. It contains 238 images of 119 people, in which half of the images are occluded images. As in Partial-REID, these images are manually cropped.

Market1501 [16] is a widely used holistic person Re-ID dataset that contains 12,936 training images, 19,732 gallery images and 3368 query images. These images were captured in Tsinghua University by six cameras.

DukeMTMC-reID [17,18] is a large holistic person Re-ID dataset. It contains 16,522 training images, 17,661 gallery images and 2228 query images. This dataset works with the Market1501 dataset to verify the performance of the SGAM on the holistic Re-ID problem.

The quality of different methods is evaluated by cumulative matching characteristic (CMC) curves and mean average precision (mAP). All the experiments were performed in a single query setting. 

### 4.2. Implementation Details

We use the ImageNet [24] pre-trained model to initialize the SGAM. In the preprocessing stage, the model uses random flipping, random cropping and random erasing [25] to enhance input images. In the training stage, the SGAM uses the standard stochastic gradient descent (SGD) optimization strategy to train the network, and the learning rate is initialized to 0.1. After 40 epochs, the learning rate decays to 0.01. To retain more information of the pre-trained model, the learning rates of all pre-trained layers are set to 0.1× of the base learning rate. The model is trained for 80 epochs.

The human semantic parsing network is trained on the Look into Person (LIP) dataset [26]. During the preprocessing stage, the network uniformly adjusts the input image size to 320 × 320. Each batch contains 16 images, and the rest of the settings are similar to those of the SGAM.

### 4.3. Results Comparison

We tested the SGAM and other classical methods on the Occluded-DukeMTMC dataset. These methods are divided into four groups. The first two groups of methods are designed for the holistic person Re-ID problem, in which the first group of methods extracts the global features of pedestrians, and the second group of methods employs horizontal segmentation, pose estimation and semantic analysis to extract local features of pedestrians. These methods do not consider the impact of occlusion. The third group of methods is designed for the partial Re-ID problem; these methods learn the ability to carry out global-part-based matching and attempt to match features of different scales. The last group of approaches is designed for the occluded Re-ID problem, in which the PGFA [12] is by far the best-performing method on Occluded-DukeMTMC.

It can be seen in Table 1 that the first two groups of methods perform poorly on the Occluded-DukeMTMC dataset. As analyzed in Figure 1, the occlusion area interferes with the features and thus affects the retrieval results. In addition, on the Occluded-DukeMTMC dataset, all probe images are not manually cropped and retain the occluded regions, so the performance of partial Re-ID methods is affected as well. Even compared with the previous best method, PGFA [12], our SGAM exceeds it by +3.4% Rank-1 accuracy. On the one hand, the SGAM cleverly employs semantic information to avoid distracting noises from occlusion areas, and on the other hand, the SGAM uses a public distance measurement strategy to align regional features, eliminating the noise in the non-shared area.

We also tested the SGAM on partial Re-ID datasets and compared it with several partial Re-ID methods. Owing to the small number of images in the two datasets, we employed the Market1501 dataset to train the SGAM. In Table 2, the SGAM’s Rank-1/Rank-3 achieve 74.3%/82.3% and 70.6%/82.4% on the two partial Re-ID datasets, which is better than all partial Re-ID methods. Even compared to the current best-performing method, PGFA, the SGAM outperforms it by +6.3% Rank-1 and +1.5% Rank-1 on Partial-REID and Partial-iLIDS. It is worth noting that the other methods [8,9,10,11,32] require manual cutting of the probe image, whereas our method does not require this process and is more practical than them.

We also verified the performance of the SGAM in the holistic person Re-ID situation. The results in Table 3 demonstrate that the performance of the SGAM is comparable to the current state-of-the-art performance. It is worth noting that the best method, PCB [2], only obtains 42.6% Rank-1 and 33.7% mAP on Occluded-DukeMTMC because the local feature comparison method that it uses is interfered with by the strong noise of the occlusion. Experimental results prove that the SGAM has good versatility in both occlusion and non-occlusion environments.

### 4.4. Ablation Study

The SGAM uses multiple strategies to deal with the occluded Re-ID problem. To study the effectiveness of each strategy, we conducted several ablation experiments on the Occluded DukeMTMC dataset. The following is the main content.

**Figure 6:**
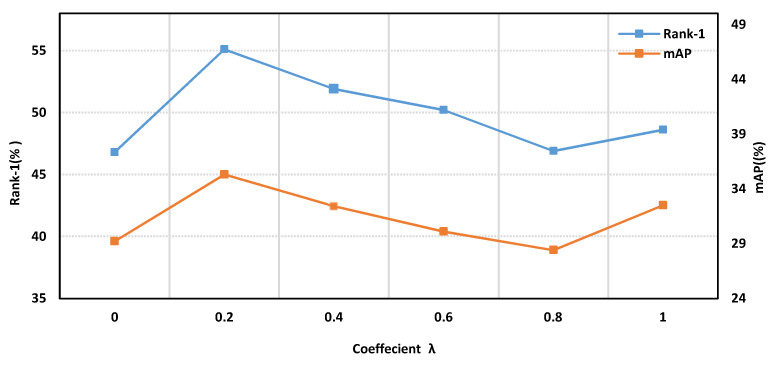
The effect of different contribution coefficients λ on model performance.

**Different Contribution Coefficient.** In order to obtain discriminative features, in the SGAM, our output features include global features and semantic-guided local features, and as shown in Equation (7), the SGAM uses λ to weigh the loss contribution of different features. To study the influence of contribution coefficients λ on the model, we set different values of λ during training and recorded the performance changes of the SGAM. The experimental results are shown in Figure 6.

The results in Figure 6 show that combining multi-granularity information is critical to the performance of the SGAM. When using only global features or local features, the SGAM cannot achieve the best performance. If only global features are used, the SGAM will be affected by noise in non-shared areas. When only local features work, the SGAM loses the global information of the image. When λ = 0.2, the SGAM has the best performance. In other experiments, we used λ = 0.2 as the default.

**The Importance of Public Areas.** To ensure that the SGAM pays attention to the visible area and focuses on the public visible area of images during retrieval, we propose a training strategy and a public distance measurement strategy. To verify the effectiveness of these two strategies, we trained two malfunctioning SGAMs for comparison:

SGAM-1 abandons the training strategy proposed in Section 3.4. In SGAM-1, all regional feature branches contribute to the final loss, even including some regions that do not exist in the image.

SGAM-2 abandons the public distance measurement strategy proposed in Section 3.3. In SGAM-2, we always accumulate the distance of all features, even including some regions that are not shared by the retrieved images.

From the results in Table 4, we can draw the following conclusions:

First, when the training strategy proposed in Section 3.4 is not used, the performance of the SGAM decreases significantly, which is easy to understand: in this case, the invisible local features also cause training losses, and the learned training features include larger sample noises. Therefore, the training strategy can guide the SGAM to focus on the visible region and learn the correct regional features.

Second, when the SGAM does not use the public distance measurement strategy, the retrieval ability of the SGAM is significantly reduced. As analyzed in Section 3.3, when the model does not filter the public visible area during retrieval, the noise of the non-public area affects the retrieval result. We thus infer that forcing the model to focus on the publicly visible area of the image through the common distance measurement strategy can effectively improve the retrieval accuracy of the SGAM.

**The Impact of Automatic Cropping Strategy.** To verify the role of the automatic cropping strategy in the SGAM, we trained an SGAM model without the automatic cropping strategy and used a ResNet50 [23] network as a baseline for comparison. From the results in Table 5, we find that after using the automatic cropping strategy, the performance of the ResNet50 [23] network is greatly improved, which shows that the strategy can help the model avoid the noise effect of the occlusion area and focus on the visible area. In addition, the performance of the SGAM model that does not use the automatic cropping strategy has dropped significantly. This is mainly because the global features and local features extracted by the SGAM are all doped with noise in the occlusion area. This interference information greatly affects the model’s retrieval ability. The experiment shows that the automatic cropping strategy can help the model extract more discriminative features and improve the model’s retrieval ability. At the same time, the strategy can be easily embedded into other pedestrian re-identification methods.

### 4.5. Visualization

**Figure 7:**
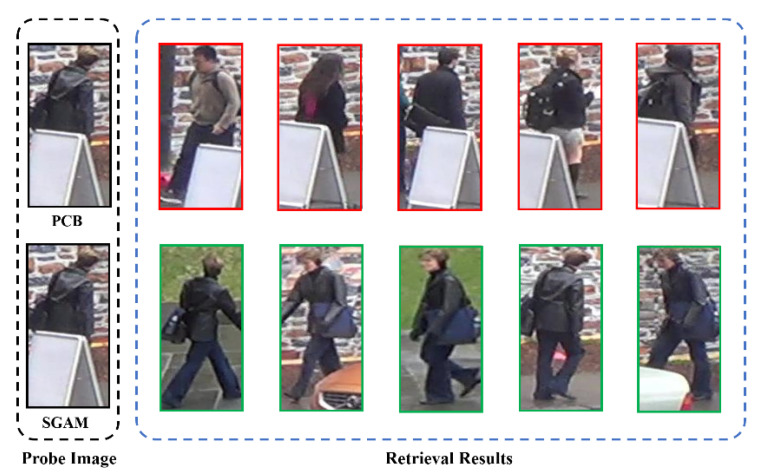
Comparison of retrieval results between PCB and SGAM.

We visualized some retrieval results of the PCB [2] method and SGAM method on the Occluded-DukeMTMC dataset. It can be observed in Figure 7 that PCB retrieves the wrong results containing similar obstacles. This is because the features extracted by PCB contain the visual information of obstacles. Therefore, in the retrieval phase, PCB considers that images containing similar obstacles are more similar to the probe image. On the contrary, our SGAM method uses semantic information as a guide to avoid the noise effect of the occlusion area. Further, the features extracted by the SGAM do not contain the information of the obstacles. In the retrieval phase, the SGAM uses the public distance measurement strategy proposed in Section 3.3 to only measure the distance of public areas between images, thus it reduces the impact of noise in non-public areas, and through this series of strategies, the SGAM successfully retrieves the correct results.

## 5. Conclusions

In this paper, we propose a model for the occluded person Re-ID problem: SGAM. The SGAM uses image semantic information as a guide to automatically crop occluded images and extracts region-level features, thus filtering out the information of the occluded areas. During retrieval, the SGAM focuses on the public visible area of images, thereby suppressing serious spatial misalignment in occluded Re-ID. We conducted extensive experiments on the SGAM on multiple datasets. The experimental results show that our method outperforms existing approaches on the occluded Re-ID problem and is also comparable to the current strongest holistic Re-ID method.

## Figures and Tables

**Table 1:** Performance comparison of different Re-ID methods on Occluded-DukeMTMC.

Methods	Rank-1 (%)	Rank-5 (%)	Rank-10 (%)	mAP (%)
DIM [27]	21.5	36.1	42.8	14.4
LOMO+XQDA [28]	8.1	17	22	5
Part Aligned [4]	28.8	44.6	51	20.2
Random Erasing [25]	40.5	59.6	66.8	30
HACNN [5]	34.4	51.9	59.4	26
Adver Occluded [29]	44.5	-	-	32.2
Part Bilinear [30]	36.9	-	-	-
FD-GAN [31]	40.8	-	-	-
PCB [2]	42.6	57.1	62.9	33.7
DSR [8]	40.8	58.2	65.2	30.4
SFR [10]	42.3	60.3	67.3	32
PGFA [12]	51.4	68.6	**74.9**	**37.3**
SGAM	**55.1**	**68.7**	74	35.3

**Table 2:** Performance comparison on Partial-REID and PartialiLIDS.

Methods	Partial-REID		Partial-iLIDS	
	Rank-1 (%)	Rank-3 (%)	Rank-1 (%)	Rank-3 (%)
MTRC [32]	23.7	27.3	17.7	26.1
AMC+SWM [9]	37.3	46.0	21.0	32.8
DSR [8]	50.7	70.0	58.8	67.2
SFR [10]	56.9	78.5	63.9	74.8
VPM [11]	67.7	81.9	65.5	74.8
PGFA [12]	68.0	80.0	69.1	80.9
SGAM	**74.3**	**82.3**	**70.6**	**82.4**

**Table 3:** Performance comparison on Market-1501 and DukeMTMC-reID.

Methods	Market1501		DukeMTMC-reID	
	Rank-1 (%)	mAP	Rank-1 (%)	mAP
BoW+kissme [16]	44.4	20.8	25.1	12.2
SVDNet [33]	82.3	62.1	76.7	56.8
PAN [17]	82.8	63.4	71.7	51.5
PAR [4]	81	63.4	-	-
Pedestrian [34]	82	63	-	-
DSR [8]	83.5	64.2	-	-
MultiLoss [35]	83.9	64.4	-	-
TripletLoss [1]	84.9	69.1	-	-
Adver Occluded [29]	86.5	78.3	79.1	62.1
APR [36]	87	66.9	73.9	55.6
MultiScale [37]	88.9	73.1	79.2	60.6
MLFN [38]	90	74.3	81	62.8
PCB [2]	92.4	77.3	81.9	65.3
PGFA [12]	91.2	76.8	82.6	65.5
VPM [11]	93	80.8	83.6	72.6
**SGAM**	91.4	77.6	83.5	67.3

**Table 4:** Experiment on the malfunctioning SGAM models.

Methods	Rank-1 (%)	Rank-5 (%)	Rank-10 (%)	mAP
SGAM	55.1	68.7	74.0	35.3
SGAM-1	47.6	58.0	63.1	29.2
SGAM-2	51.8	62.8	68.1	32.2

**Table 5:** The impact of the automatic cropping strategy on model performance.

Method	Rank-1	Rank-5	Rank-10	mAP
ResNet50	39.5	57.2	63.7	27.2
ResNet50+crop	48.2	65.9	73.1	32.5
SGAM (no crop)	44.8	61.3	68.6	35.0
SGAM	**55.1**	**68.7**	**74.0**	**35.3**
